# Prolactin-elevating Antipsychotics: Anti-hero or Savior?

**DOI:** 10.31083/AP38785

**Published:** 2025-02-28

**Authors:** Chong Guan Ng, Benedict Francis

**Affiliations:** ^1^Department of Psychological Medicine, Faculty of Medicine, University of Malaya, 50603 Kuala Lumpur, Malaysia

Prolactin is a key hormone responsible for lactation, breast development, and other actions needed to maintain homeostasis. Prolactin has had a thorny relationship with cancer development, particularly breast cancer. To date, there is conflicting evidence that prolactin elevation is directly associated with the increased risk of breast cancer. A recent meta-analysis concluded that the correlation between plasma prolactin and breast cancer is at best circumstantial with new evidence pointing towards the role of overexpression of local breast tissue secreting prolactin as the culprit [1]. One study found that although the risk of breast cancer was higher than usual in individuals on prolactin-elevating antipsychotics, causal linkage cannot be demonstrated due to the presence of many confounding factors [2].

Prolactin-elevating antipsychotics such as amisulpride, paliperidone, risperidone, and typical antipsychotics have traditionally been vilified for increasing breast cancer risk [3, 4]. However, tumorigenesis is multifactorial and complex, and may be caused by a plethora of factors such as smoking, malignancies, and other endocrinological causes [5, 6, 7]. Paliperidone, for example, is known to cause a higher quantum of prolactin increase compared with other antipsychotics due to its strong and sustained D2 blockade in the tuberoinfundibular pathway [8].

Rahman *et al*. [3] (2022) studied the relationship between antipsychotic-induced hyperprolactinemia and breast cancer. This epidemiological study used the MarketScan databases in the United States. The study subjects consisted of young women up to 64 years old with an outpatient prescription drug claim for an antipsychotic, anticonvulsant, or lithium from January 1, 2007 to June 30, 2016. Antipsychotic prescriptions were stratified based on their propensity to elevate prolactin. Invasive breast cancer was identified using International Classification of Diseases Ninth and Tenth Revisions, Clinical Modification (ICD-9/10-CM) codes, CPT-4 codes, or evidence of surgical treatment or chemotherapy. The results showed that exposure to any antipsychotics was independently associated with a 35% increased risk of breast cancer (adjusted hazard ratio [aHR], 1.35; 95% confidence interval [CI], 1.14–1.61). Category 1 drugs (prolactin-elevating antipsychotics including paliperidone) were associated with a 62% increased risk (aHR, 1.62; 95% CI, 1.30–2.03). However, there are several limitations and flaws in the study design. Firstly, there was insufficient control of potential confounders such as menopausal status, family history, and alcohol consumption. Secondly, the comparators used were inappropriate as antipsychotics were compared against anticonvulsants and lithium. Lastly, the incidence proportion of breast cancer in patients on antipsychotics was not significantly different from the general population [3].

Subsequent epidemiologic studies have produced contrasting findings. A retrospective observational cohort study conducted by Kern *et al*. [9] and published in 2024 utilized the MarketScan Medicaid database consisting of 33 million users. This study examined the association between antipsychotic-induced prolactin increase and breast cancer risk, using two methods of defining breast cancer: the Rahman criteria and the Nattinger Algorithm (considered the gold standard for diagnosis). This study, perhaps unsurprisingly, found that there was no statistically significant association between prolactin-exposure and the increased risk of breast cancer (hazard ratio, 0.96 [95% CI, 0.62–1.48]–1.28 [0.40–4.07]) [9].

The risk of breast cancer linked to risperidone intake was investigated in a different retrospective cohort study. The study identified all women who were 18 years of age or older who started treatment with any antipsychotic between July, 2000 and December, 2011 by using information from Taiwan’s National Health Insurance Research Database (NHIRD), which records claims from mandated universal health insurance. The Taiwan Cancer Registry and the NHIRD Registry of Catastrophic Illness were used to identify breast cancer cases. Risperidone was not linked to an increased risk of breast cancer when compared with other atypical or conventional antipsychotics among the 233,237 women who were included in the study. The aHRs for other atypical and typical antipsychotics were 1.07 (95% CI, 0.95–1.22) and 1.13 (95% CI, 0.98–1.29) [10]. In 2018, Tsai *et al*. [10] conducted a systematic review using data from 11 relevant studies with 1,499,001 participants to examine the epidemiological evidence of antipsychotic exposure and breast cancer risk. The findings revealed a relatively low heterogeneity in the incidence of breast cancer (odds ratio (OR), 1.13; 95% CI, 0.97–1.31) between prolactin-increasing and prolactin-sparing antipsychotics. The research concluded that there are currently insufficient data to support the hypothesis that hyperprolactinemia brought on by antipsychotics increases the risk of breast cancer [11].

In the authors’ opinion, prolactin-elevating antipsychotics may have benefits over prolactin-sparing antipsychotics in treating schizophrenia patients with prominent aggression and positive symptoms. The former group of antipsychotics possesses strong affinity and low intrinsic activity at D2/D4 receptors. The more potent net dopaminergic blockade is more beneficial in alleviating aggression displayed by patients with schizophrenia [12, 13]. The superior tolerability profile of prolactin-sparing antipsychotics, such as partial agonists, may be useful for some patients although inadequate for highly agitated and aggressive patients. Thus, proper patient selection is imperative.

In conclusion, it is crucial to balance the efficacy and safety of prolactin-elevating antipsychotics when treating patients with schizophrenia. Shared decision-making should be the cornerstone of treatment, involving effective communication and dialogue with patients and their families to align treatment expectations and achieve favorable outcomes. While prolactin may be a double-edged sword in managing schizophrenia, judicious selection of antipsychotics and optimal risk-benefit assessments will best serve the patient’s needs. Clinicians must be mindful of the fact that breast cancer is complex and multifactorial.
